# A Nelore Cattle Structural Variant Catalog

**DOI:** 10.1002/age.70225

**Published:** 2026-10-18

**Authors:** Thales de Lima Silva, Alexander S. Leonard, Pablo Augusto de Souza Fonseca, Haroldo Henrique de R. Neves, Alexandre M. Ruiz, Lucas Tassoni Andrietta, Gerardo A. Fernandes Júnior, Júlio Cesar de C. Balieiro, Hubert Pausch, Roberto Carvalheiro, Ricardo Vieira Ventura

**Affiliations:** ^1^ Department of Animal Nutrition & Production, School of Veterinary Medicine and Animal Science University of São Paulo Pirassununga Brazil; ^2^ Animal Genomics, ETH Zürich Zürich Switzerland; ^3^ Instituto de Ganadería de Montaña CSIC – Universidad de León Grulleros León Spain; ^4^ GenSys Consultores Associados Jaboticabal Brazil; ^5^ Department of Animal Science Universidade Estadual Vale Do Acaraú Sobral Brazil; ^6^ Department of Animal Science, School of Agricultural and Veterinarian Sciences São Paulo State University Jaboticabal Brazil; ^7^ Commonwealth Scientific and Industrial Research Organization Hobart Tasmania Australia

**Keywords:** *Bos indicus*, Oxford Nanopore long‐read sequencing, tropical adaptation

## Abstract

Structural variants (SVs) contribute more nucleotide divergence between mammalian genomes than single nucleotide polymorphisms, yet no population‐scale, long‐read‐validated SV catalog has been available for *Bos indicus* cattle. Nelore, the most numerically important indicine breed worldwide, diverges substantially from the taurine‐derived reference genome used for most bovine SV discovery, leaving its structural variation largely uncharacterized. Long‐read sequencing of 20 Nelore bulls with two complementary callers (Sniffles2, SVIM) yielded 82 581 dual‐caller concordant SVs, of which 74 222 insertions and deletions passed independent read‐based validation. Ensembl VEP annotation identified 561 HIGH‐impact variants (0.75% of the catalog) across 7040 protein‐coding genes. Only 22 894 SVs (30.7%), including 147 of 561 HIGH‐impact variants (26.2%), could be tagged by an existing BovineHD SNP (*r* ^2^ ≥ 0.8), showing that routine array genotyping captures a minority of this variation. Intersection with an indicine‐ancestry SNP map identified 6114 SVs (8.2%) enriched in *Bos indicus* , including 61 HIGH‐impact variants overlapping genes with plausible roles in immune/inflammatory defense and oxidative‐stress response, providing a resource and candidate variant set for genomic selection and tropical‐adaptation studies in indicine cattle.

## 1 Introduction

Structural variants (SVs) are segments of DNA > 50 bp that differ in copy number or orientation between individuals or populations (Feuk et al. 2006). These variants comprise deletions, insertions, duplications, and inversions, collectively representing a larger fraction of nucleotide differences across genomes than single nucleotide polymorphisms (SNPs) (Sudmant et al. 2015). SV discovery and characterization have been historically limited by the reliance on short‐read sequencing technologies, which lack the sensitivity to reliably detect breakpoints in repetitive or complex regions (Mahmoud et al. 2019). Long‐read sequencing platforms have fundamentally changed this landscape, enabling researchers to resolve SVs at breakpoint resolution across entire genomes (Sedlazeck et al. 2018).

In livestock, and particularly in cattle ( *Bos taurus* and *Bos indicus* ), structural variation has been recognized as an important source of phenotypic diversity (Bickhart and Liu 2014; Nguyen et al. 2023): specific deletions carry antagonistic effects on fertility and milk yield (Kadri et al. 2014), complex structural variants upstream of *KIT* drive coat depigmentation in white‐headed taurine breeds (Milia et al. 2024), and their broader influence on production traits, disease susceptibility, and fertility has been documented (Bickhart and Liu 2014). However, the available SV catalogs for cattle have been generated primarily from short‐read sequencing data in taurine breeds (Bhati et al. 2023; Boussaha et al. 2015; Grant et al. 2024; Lee et al. 2023). This constrains resolution for complex rearrangements and leaves indicine populations substantially undercharacterized.

Nelore cattle ( *Bos indicus* ), which account for a large proportion of the Brazilian beef cattle population, represent a unique genetic background with substantial divergence from taurine ( *Bos taurus* ) cattle (Porto‐Neto et al. 2013). This divergence encompasses both SNP and SV polymorphisms that remain largely unexplored beyond CNV surveys based on SNP arrays and short‐read data (da Silva et al. 2016). Studies in East Asian indicine cattle have revealed population‐specific SVs contributing to local environmental adaptation and disease resistance (Xia et al. 2023). High‐quality indicine genome assemblies (Leonard et al. 2022) and multi‐species pangenomes incorporating indicine sequence (Bortoluzzi et al. 2024) have advanced the genomic resources for comparisons between *Bos taurus* and *Bos indicus* . However, no population‐scale long‐read SV catalog exists for Nelore or other South American indicine populations. Understanding the landscape of structural variation in Nelore populations is essential for interpreting genome‐wide association studies, designing genomic breeding programs, and exploiting the full spectrum of variation for genetic improvement.

Recent advances in long‐read sequencing, particularly Oxford Nanopore Technologies, have made high‐depth, population‐scale SV discovery tractable (Liu 2025; Nguyen et al. 2023). Cohort‐scale long‐read sequencing studies in taurine cattle have demonstrated that SVs are substantially enriched among molecular QTL relative to SNPs (Mapel et al. 2026), and that breed‐specific pangenome graph approaches identify over 10 000 additional SVs per sample compared to short‐read methods while improving association mapping (Yang et al. 2026). Bovine super‐pangenomes incorporating taurine and indicine assemblies have further demonstrated that SV counts and tandem‐repeat landscapes differ substantially between *Bos taurus* and *Bos indicus* , including a VNTR locus with allele‐specific expression confirmed in a Nelore individual (Leonard et al. 2023). However, the interpretation of SVs called from long‐read data depends critically on validation, as callers differ in sensitivity and specificity across variant types and genomic contexts (Gao et al. 2022; Mahmoud et al. 2019). Read‐based validation approaches provide a direct, independent means to filter high‐confidence SVs (Zheng and Shang 2024), while linking SVs to existing SNP genotyping arrays (such as the BovineHD BeadChip) enables their incorporation into routine genomic evaluations without requiring additional sequencing.

Here, we address this gap by establishing the first dual‐caller, read‐validated population‐level SV catalog for Nelore cattle using ONT long‐read sequencing. We demonstrate that the indicine SV landscape is substantially distinct from taurine reference‐anchored resources, identify a subset of functionally consequential variants enriched in *Bos indicus* , and show that existing SNP chip tools capture only a fraction of this variation.

## 2 Material and Methods

Approval from the Ethics Committee on Animal Use (CEUA) of the School of Veterinary Medicine and Animal Science (FMVZ) from the University of São Paulo (USP) was recorded under the process CEUA N° 2600190922.

### 2.1 Animal Selection

Whole‐genome sequencing (WGS) data were generated from semen samples of 20 Nelore bulls ( *Bos indicus* ) as part of the COWADAPT project using Oxford Nanopore Technologies (ONT). A careful selection of bulls representative of the Nelore cattle population in Brazil was carried out using data from different programs that comprise the Aliança Nelore database (GenSys 2019). Several criteria were used to establish the final ranking, which sought to encompass informativeness in the Aliança database and potential contribution in terms of genetic diversity (SI Appendix, Table S1). The following criteria were considered: the genomic relatedness coefficient between bulls; number of offspring (and herds) with controlled phenotype in the Aliança database; ranking of the bull in analysis of marginal contributions (Boichard et al. 1997) considering the pedigree of the Aliança database; cluster referring to the partition with 10 groups, obtained by hierarchical grouping, based on the genomic relationship matrix among 2544 most informative bulls genotyped in the Aliança database.

The ranking was determined through an iterative process. Starting from an initial ranking considering marginal contributions, number of offspring at weaning in the Aliança Nelore program, and number of herds with bull offspring in the Aliança Nelore program, the most informative animal was selected. Then, the ranking was recursively updated, penalizing individuals from the same cluster of animals previously selected. An alternative ranking was also generated through a similar process but considering only the average relatedness to the previously selected animals. The “Final Rank” considered these two rankings and additional constraints regarding lineage/cluster to define the top 20 animals.

### 2.2 Long Read Sequencing

High‐molecular‐weight DNA was extracted from frozen semen following the protocol of Denis et al. (2024), and DNA concentration was quantified using a Qubit fluorometer. Libraries were prepared following the V14 Ligation Sequencing Kit (SQK‐LSK114) protocol and sequenced on the PromethION platform (R10.4.1 pores) in 72‐h runs. For each sample, ~200 ng DNA was loaded onto a FLO‐PRO114M flow cell, followed by a mid‐run wash (after ~25 h) and reloading with an additional 200 ng. Real‐time basecalling was performed using the fast accuracy model (FAST), and reads were subsequently re‐basecalled using the super accuracy model (SUP) in Dorado (https://github.com/nanoporetech/dorado) v0.7.2.

Adapter and barcode sequences were removed using Porechop (https://github.com/rrwick/Porechop) (v0.2.4.; Wick 2017). Reads were subsequently filtered with SeqKit2 v2.8.2 (Shen et al. 2024) using the standard ONT quality threshold (>Q10 for the SUP model). Reads were aligned to the bovine reference genome ARS‐UCD2.0 (https://www.ncbi.nlm.nih.gov/datasets/genome/GCF_002263795.3/) (GCA_002263795.4) using Minimap2 (v2.30‐r1287; Li 2018, 2021) with the *‐ax map‐ont* preset. BAM files were coordinate‐sorted and indexed using SAMtools (v1.23.1, Danecek et al. 2021). Mean per‐sample sequencing depth was 22.11× (range: 10.48–39.51×; Table S2), assessed with Mosdepth (v0.3.10; Pedersen and Quinlan 2018).

### 2.3 SV Calling, Merging, and Concordance Filtering

The SVs were called from the alignments using two callers: Sniffles2 (v2.7.2; Smolka et al. 2024) and SVIM (v2.0.0; Heller and Vingron 2019). Both callers were run with default parameters on all 20 samples. Both Sniffles2 and SVIM use split‐read alignments and high‐mismatch regions as signatures to identify SVs, and their combined use was intended to increase confidence in downstream analyses by leveraging complementary detection strategies. SV types targeted in this study included deletions (DEL), insertions (INS), duplications (DUP), and inversions (INV). Translocations, although detected in the initial SV discovery phase by Sniffles2 and SVIM, were excluded from subsequent analyses because they cannot be independently validated with SVvalidation (Zheng and Shang 2024).

Per‐sample VCF files from Sniffles2 and SVIM were first restricted to autosomal chromosomes using BCFtools (v1.23.1; Danecek et al. 2021). Sex chromosomes were excluded because all animals were males, and our pipeline assumes diploid genotypes, so SVs on the sex chromosomes remain uncharacterized in Nelore. Their analysis will require haploid‐aware genotyping and a Y reference representative of indicine haplogroups (Chen et al. 2023). Before merging, SVs shorter than 50 bp were discarded and those supported by fewer than five reads were flagged and excluded as low‐confidence calls. Additionally, SVs located in regions where read depth exceeded five times the sample‐level mean BAM coverage were removed as likely artifacts of repetitive or collapsed genomic regions (Zheng and Shang 2024). Per‐sample concordant SVs were then identified using SURVIVOR (v1.0.7; Jeffares et al. 2017) with a maximum breakpoint distance of 1000 bp, requiring support from both callers (minimum supporting callers = 2), and enforcing both strand consistency and SV type matching. This dual‐caller intersection strategy retains only SVs independently supported by both Sniffles2 and SVIM, reducing the false‐positive rate inherent to individual long‐read SV callers (Gao et al. 2022; Liu 2025).

The resulting per‐sample concordant VCF files were subsequently merged across all 20 animals to produce a non‐redundant population‐level SV catalog. SURVIVOR merge was applied twice with breakpoint distance thresholds of 500 bp and 1000 bp, requiring concordance across at least two samples, with strand and SV‐type consistency enforced in both runs. The two outputs were compared to assess the robustness of variant retention to this parameter: a difference of less than 10% in total SV count between the two runs was considered indicative of a stable call set, and the 1000‐bp threshold output was retained for downstream analyses. SV type composition of the final catalog was assessed using BCFtools *query*.

### 2.4 Read‐Based SV Validation

Concordant SVs were subjected to read‐based validation using SVvalidation (Zheng and Shang 2024). BAM files from 20 samples were indexed with SAMtools prior to validation. For each sample, SVvalidation was executed with a BED file of SV calls as input, producing per‐sample output files in which each SV was classified as homozygous (*homo_*), heterozygous (*heter_*), or false positive (*false_*). This classification reflects the read‐level evidence spanning each candidate breakpoint: calls supported by SV‐consistent alignments in all informative reads were classified as homozygous, calls supported in only a subset of reads were classified as heterozygous, and calls for which the read alignments provided no support for the reported variant at that location were classified as false positive (Zheng and Shang 2024). SVs classified as either homozygous or heterozygous were considered validated in that sample.

We aggregated the sample validation results against the original SV reference set to assess cross‐sample support. SVs were matched across files using a bin‐based coordinate key with a tolerance of 200 bp on both start and end positions and classified by SV type. For each SV in the reference set, the script counted the number of samples in which it was independently validated and recorded the contributing sample identifiers. SVs validated in at least two independent samples were retained for downstream analyses, as this threshold provides cross‐sample corroboration while accounting for population‐level variation in SV segregation. Retained SVs were intersected against the merged VCF using BEDTools *intersect* (v2.31.2; Quinlan and Hall 2010) to produce the final validated SV callset.

SVvalidation determines deletion support from the presence of an explicit deletion (D) operation in the CIGAR string of aligned reads. For small deletions (< 500 bp) in tandem‐repeat‐ and homopolymer‐rich regions of the bovine genome, long‐read aligners frequently resolve ambiguous alignment paths by representing the variant as a mismatch or a soft‐clipped read end rather than an explicit D operation (Li 2018), causing SVvalidation to systematically under‐detect these variants. To address this limitation, DELs shorter than 500 bp were instead validated using the DV field (number of reads supporting the alternate allele) reported in the per‐sample Sniffles2 VCF, computed independently of downstream alignment representation. A DEL < 500 bp was retained in the final catalog if it had DV ≥ 5 in at least two independent animals. DELs ≥ 500 bp and all insertions retained the original SVvalidation‐based criterion described above.

### 2.5 Functional Annotation With VEP

Validated SVs were functionally annotated using the Ensembl Variant Effect Predictor (VEP v111; McLaren et al. 2016), run in offline mode with the *Bos taurus* cache (Ensembl release 111, assembly ARS‐UCD2.0). The VEP annotation was performed with *‐‐everything*, *‐‐pick*, *‐‐symbol*, *‐‐check_existing*, and *‐‐distance* 1000, assigning consequences up to 1000 bp upstream and downstream of SV breakpoints. The *‐‐pick* flag was used to retain a single consequence per variant, selected according to VEP's ranked criteria prioritizing the most severe effect. Annotations for each SV contained: chromosomal coordinates, SV type, SV length, predicted consequence, gene symbol, Ensembl gene and feature identifiers, feature type, biotype, exon/intron context, HGVSc notation, functional impact category (HIGH, MODERATE, LOW, or MODIFIER), distance to nearest feature, strand, and existing variant identifiers. SVs with no overlapping or proximal annotation within the defined distance were retained in the output with missing values for consequence fields. SVs classified as HIGH impact were extracted for prioritization in downstream biological interpretation.

### 2.6 SNP Calling From Aligned ONT Reads

Per‐sample SNP variants were called from the coordinate‐sorted BAM files using Clair3 (v1.0.10; Zheng et al. 2022) with the r1.041_e82_400bps_sup_v500 model, optimized for R10.4.1 flowcell data basecalled with the SUP model. The resulting per‐sample GVCF files were compressed with bgzip (v1.19.1; Danecek et al. 2021). All 20 per‐sample GVCFs were combined with GATK *CombineGVCFs* and jointly re‐genotyped with GATK *GenotypeGVCFs* (GATK v4.0; McKenna et al. 2010). SNPs were extracted with GATK *SelectVariants* (*‐‐select‐type‐to‐include SNP*), excluding indels, and quality‐filtered with GATK *VariantFiltration* using published hard‐filter expressions (Yin et al. 2025). Variants passing all quality thresholds were retained with BCFtools *view* (*‐f PASS*) and the callset was restricted to the 29 autosomes with BCFtools *view ‐r*.

### 2.7 Extraction of SNPs Matching BovineHD Chip Positions

Whole‐genome SNP calls from WGS data were filtered to retain only positions overlapping the Illumina BovineHD BeadChip (777 962 loci). Chip coordinates were first lifted to ARS‐UCD2.0. Biallelic SNPs at chip positions were extracted with BCFtools *view* (*‐m2 ‐M2*, *AC* > *0*). Quality filters were then applied with PLINK2 (Chang et al. 2015): minor allele frequency ≥ 0.05, genotype missingness ≤ 10%, and Hardy–Weinberg equilibrium *p* > 1 × 10^−6^. The BovineHD BeadChip was designed primarily from taurine cattle populations (Matukumalli et al. 2009), and a substantial proportion of its 777 962 positions is expected to be monomorphic or at reduced minor allele frequency in Nelore. The MAF ≥ 0.05 threshold was therefore applied to retain positions with demonstrated polymorphism in the present Nelore cohort, partially mitigating the ascertainment bias of using a taurine‐designed array in an indicine population. After all QC filters, 479 252 SNPs (61.6% of chip positions) were retained as informative for linkage disequilibrium (LD) analysis in this cohort.

### 2.8 Linkage Disequilibrium Analysis and Tag SNP Identification

To enable proxy‐based genotyping of detected SVs in routine genomic evaluations, we computed pairwise LD between SVs and BovineHD SNPs. SV genotypes were first converted from multi‐allelic (0/0, 0/1, 1/1) to biallelic encoding (REF = A, ALT = T). Converted SV VCFs were merged with the BovineHD SNP VCF using BCFtools *concat* and converted to PLINK2 binary format. Pairwise unphased *r* ^2^ was computed with PLINK2 using a 100‐kb window and reporting all pairs with *r* ^2^ ≥ 0.8 (*‐‐r2‐unphased ‐‐ld‐window‐kb* 100 *‐‐ld‐window‐r2* 0.8). SVs were encoded as single biallelic markers at their start coordinate, and SV length was not considered. For deletions, SNPs located inside the deleted interval are hemizygous or absent in carriers, so their *r* ^2^ reflects genotype concordance rather than haplotype‐based LD. We therefore quantified how many tag SNPs fell inside deletion intervals. For each SV, the SNP with the highest *r* ^2^ was designated as its tag SNP, subject to a minimum threshold of *r* ^2^ ≥ 0.8 (Grant et al. 2024).

A final catalog was assembled by integrating VEP functional annotations, tag SNP data, and per‐SV read support counts from SVvalidation, matched by a composite key of chromosome, position, and SV type to prevent redundant cross‐matches at shared genomic positions. The catalog reports SV ID, tag SNP identifier, *r* ^2^, physical distance between SV and tag SNP, annotated gene, functional consequence, and predicted impact level. A high‐impact subset was defined as SVs with HIGH predicted VEP impact. Among these, SVs carrying at least one qualifying BovineHD tag SNP (*r* ^2^ ≥ 0.8) were further identified to quantify the proportion of functionally significant SVs tractable for proxy‐based genotyping with existing array data.

### 2.9 Indicine‐Specificity Analysis

To characterize the ancestral context of detected SVs, we intersected SV loci with a genome‐wide SNP map of *Bos indicus* content derived from Kasarapu et al. (2017). That resource estimated the contribution of individual SNPs to the *Bos indicus* genome content by subjecting PCA‐derived SNP weights to Normal mixture modelling across 18 363 cattle genotyped for 729 068 SNPs, with 31% of SNPs assigned to the *Bos indicus* component. The indicine‐versus‐taurine ancestry signal used here is independent of the ARS‐UCD2.0 reference sequence: the reference provides only a shared coordinate framework, while the indicine‐versus‐taurine classification derives entirely from this population‐genetic analysis of allele frequencies across those 18 363 animals. Each SV was assigned indicine ancestry indirectly, through the SNPs that overlap or flank it. Because nearby SNPs and genomic variants tend to be inherited together in the same haplotype block, when most of the SNPs within an SV window carry a high probability of *Bos indicus* origin, the SV is inferred to reside on an indicine‐derived genomic background. This is therefore an indirect inference based on the ancestry of nearby SNPs, not direct proof that the SV allele itself originated in *Bos indicus* . Because the original SNPmap was published in UMD3.1 coordinates, SNP positions were remapped to ARS‐UCD2.0 using a chip‐based coordinate lookup table (HDmap_UMD_ARS) before intersection. Each overlapping SNP was classified as indicine‐specific if its posterior probability of *Bos indicus* origin (Pr_Indicine) exceeded a threshold of 0.5. SVs were then assigned to one of four categories: *i* *ndicine‐enriched* (≥ 75% of overlapping SNPs classified as indicine‐specific), *taurine‐enriched* (≤ 25%), *Mixed*, or *No_overlap* (no SNPs from the map within the window). Summary statistics reported per SV included the total number of overlapping SNPs, the count of indicine‐specific and taurine SNPs, the indicine fraction, and the mean and maximum Pr_Indicine values.

## 3 Results

Whole‐genome sequencing of 20 Nelore bulls on the PromethION platform yielded between 43 and 121 Gb of raw data per sample (mean: 57.2 Gb), corresponding to sequencing depths of 16–45× against the ARS‐UCD2.0 reference genome (~2.7 Gb). After re‐basecalling with Dorado v0.7.2 (SUP model) and quality filtering (>Q10), a minimum of 31.4 Gb of high‐quality reads was retained per sample. The mean N50 read length across all samples was 10.44 kb (range: 8.4–13.7 kb), consistent with high‐molecular‐weight DNA extraction from semen.

### 3.1 SV Calling, Concordance Filtering, and Population‐Level Catalog

SV calling with Sniffles2 and SVIM yielded, in the merged dataset of 20 samples, 97 353 and 99 278 raw calls, respectively, spanning deletions, duplications, insertions, and inversions (Table 1). After per‐sample inter‐caller concordance filtering, 82 581 SVs were retained as dual‐caller concordant calls across all 29 autosomes prior to read‐based validation. The inter‐caller concordance rate was highest for DEL and INS (87.8% and 83.5% of Sniffles2 calls retained, respectively), while DUP and INV showed markedly lower inter‐caller agreement (12.6% and 37.4%, respectively).

**TABLE 1:** Number of each variant type detected in the Nelore dataset by Sniffles2 and SVIM.

Type	Sniffles	SVIM	Shared^a^
DEL	44 338	45 133	38 928
DUP	485	958	61
INS	51 995	52 436	43 392
INV	535	751	200
ALL	97 353	99 278	82 581

^a^ Shared: SVs called concordantly by both Sniffles2 and SVIM and retained after per‐sample concordance filtering.

### 3.2 Read‐Based Validation

Of the 82 581 SVs in the merged concordant call set, 78 556 (95.13%) were read‐validated in at least one sample using the dual‐criterion approach, SVvalidation for INS and DELs ≥ 500 bp, and Sniffles2 DV read support (DV ≥ 5) for DELs < 500 bp, 76 052 (92.09%) were validated in at least two independent samples, and a robust call set of 58 622 SVs (70.99%) in five or more samples. Across all 20 animals, genotype‐level classification yielded 172 619 and 606 867 sample‐level observations for homozygous and heterozygous calls, respectively (Figure 1).

**FIGURE 1:**
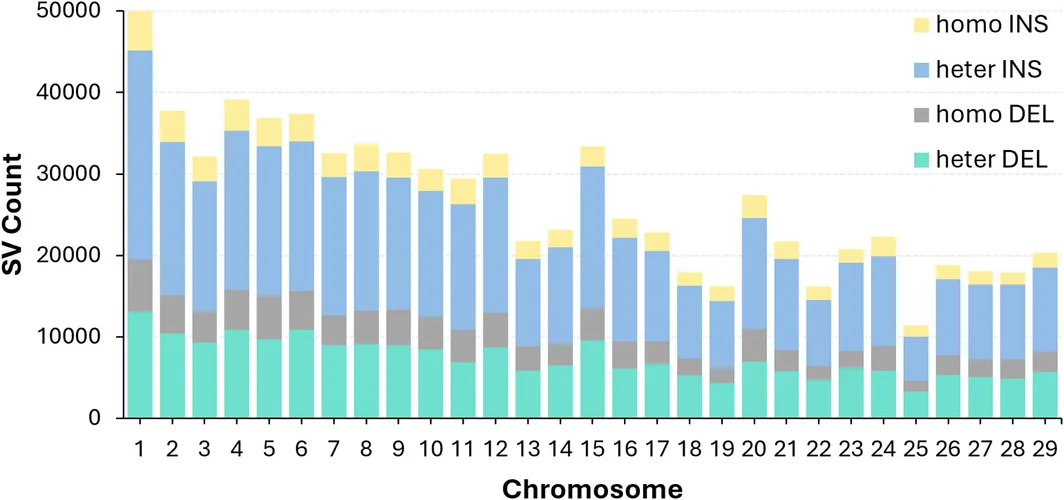
Distribution of validated SV categories by genotype classification per sample. Per‐chromosome counts for each category are reported in Table S3.

Validation rates differed markedly by SV type: DEL showed a low read‐validation rate of 37.33% when assessed by SVvalidation alone, while INS achieved the highest read‐validation rate of 98.71%. Size‐stratified analysis showed that the low DEL rate was concentrated in variants < 500 bp (Table S4). Applying an alternative, Sniffles2 read‐support‐based criterion (DV ≥ 5 in at least two animals) to this size class recovered most of these DELs, raising the overall DEL validation rate to 81.4% (31 680 of 38 928). INS validation was essentially unchanged by this revision. No DUP, INV, or TRA calls were read‐validated in the SVvalidation pipeline. However, the 61 concordance‐filtered DUP and 200 INV calls are retained in the complete catalog as unvalidated reference entries (Table 2, Read‐validated = No). The analysis‐ready SV set used for all subsequent functional and population‐genomic analyses comprised the 74 222 read‐validated INS and DEL variants (Table 2). Size profiles also differed by SV type: DEL length was concentrated in short size classes (median 0.1 kb), whereas INS spanned modestly longer lengths (median 0.2 kb); the concordance‐filtered DUP and INV calls, although not read‐validated, showed longer and more dispersed size distributions (median 6.8 kb and 1.1 kb, respectively) (Figure 2).

**TABLE 2:** Read‐based validation summary by structural variant (SV) type.

SV type	Input	Validated	Validation rate (%)	Catalog entries^a^	Read‐validated
DEL	38 928	31 680	81.38	31 680	Yes
INS	43 392	42 832	98.71	42 542	Yes
DUP	61	0	0.00	61	No
INV	200	0	0.00	200	No
Total	82 581	74 512	90.24	74 483	

^a^ Reflects all SVs retained in the complete population‐level catalog, which includes the concordance‐filtered DUP and INV calls even though these types were not read‐validated by SV validation (Read‐validated = No). DEL Catalog Entries and Validation Rate reflect the dual‐criterion approach described in Methods (SVvalidation for DELs ≥ 500 bp; Sniffles2 DV ≥ 5 in ≥ 2 animals for DELs < 500 bp).

**FIGURE 2:**
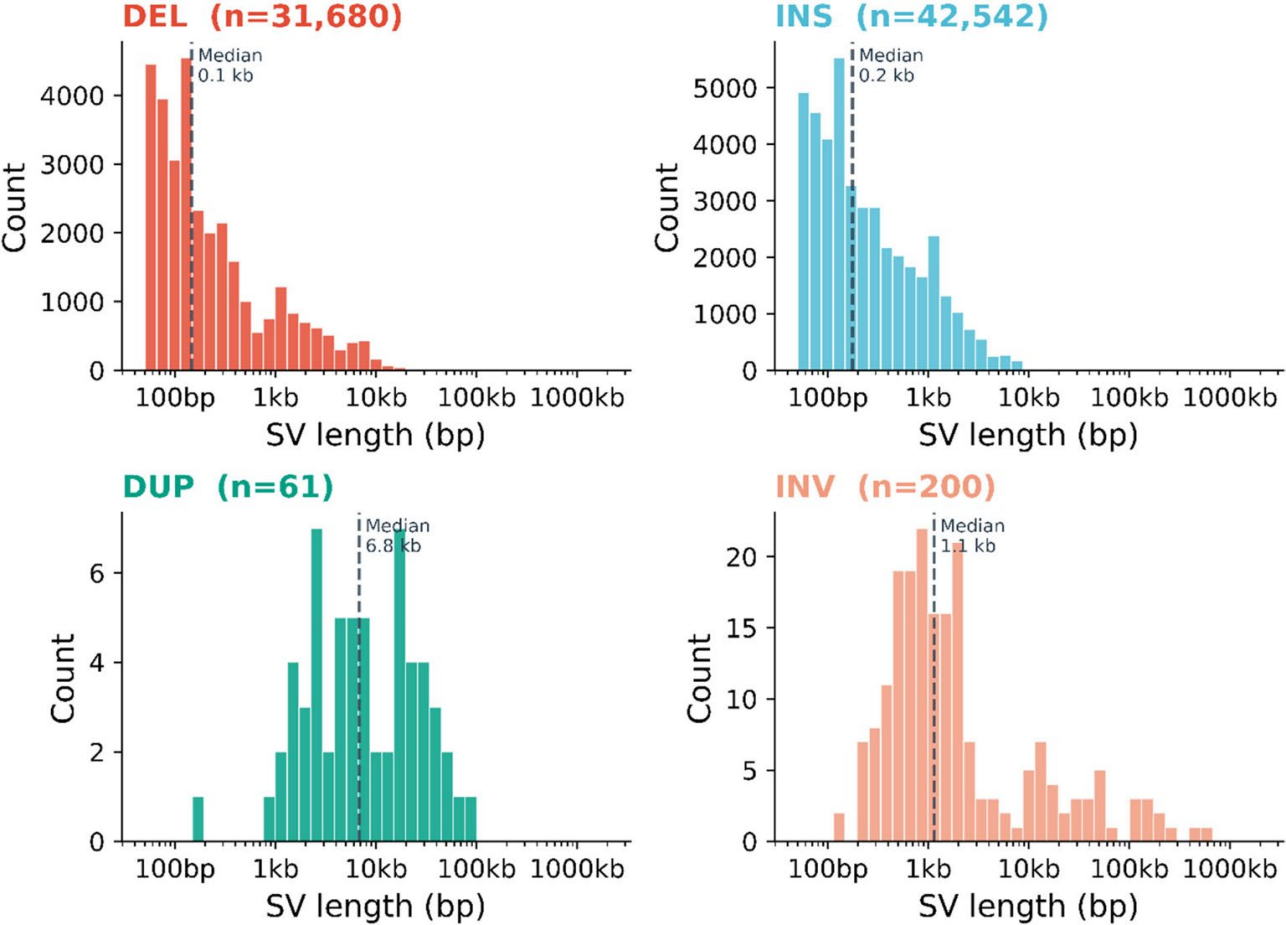
SV size distribution by type. Length distribution (bp, log10‐scaled x‐axis) of read‐validated SVs, plotted separately. Dashed vertical lines mark the median length for each SV type.

### 3.3 Functional Annotation

Functional annotation of read‐validated SVs with Ensembl VEP (ARS‐UCD2.0 cache, distance ±1 kb) assigned consequences to 74 483 variants overlapping or proximal to 7040 protein‐coding genes. The most frequent consequence category was intergenic_variant (38 077 SVs; 51.12%), followed by intron_variant (26 163 SVs; 35.13%) (Figure 3A). Under the impact tier, 73 758 SVs were classified as MODIFIER, 98 were assigned to MODERATE impact and 561 SVs (0.75% of the catalog) were assigned HIGH impact, indicating predicted disruption of gene function (Figure 3B). Within this set, VEP assigned the transcript_ablation consequence to 158 SVs (0.21% of the catalog) whose breakpoints span the entire coding sequence of a transcript (Figure 3A). HIGH‐impact SVs were distributed across all autosomes, with BTA15, BTA18, BTA08 and BTA23 showing the highest per‐chromosome counts (26–32 SVs per chromosome) (Figure 3C).

**FIGURE 3:**
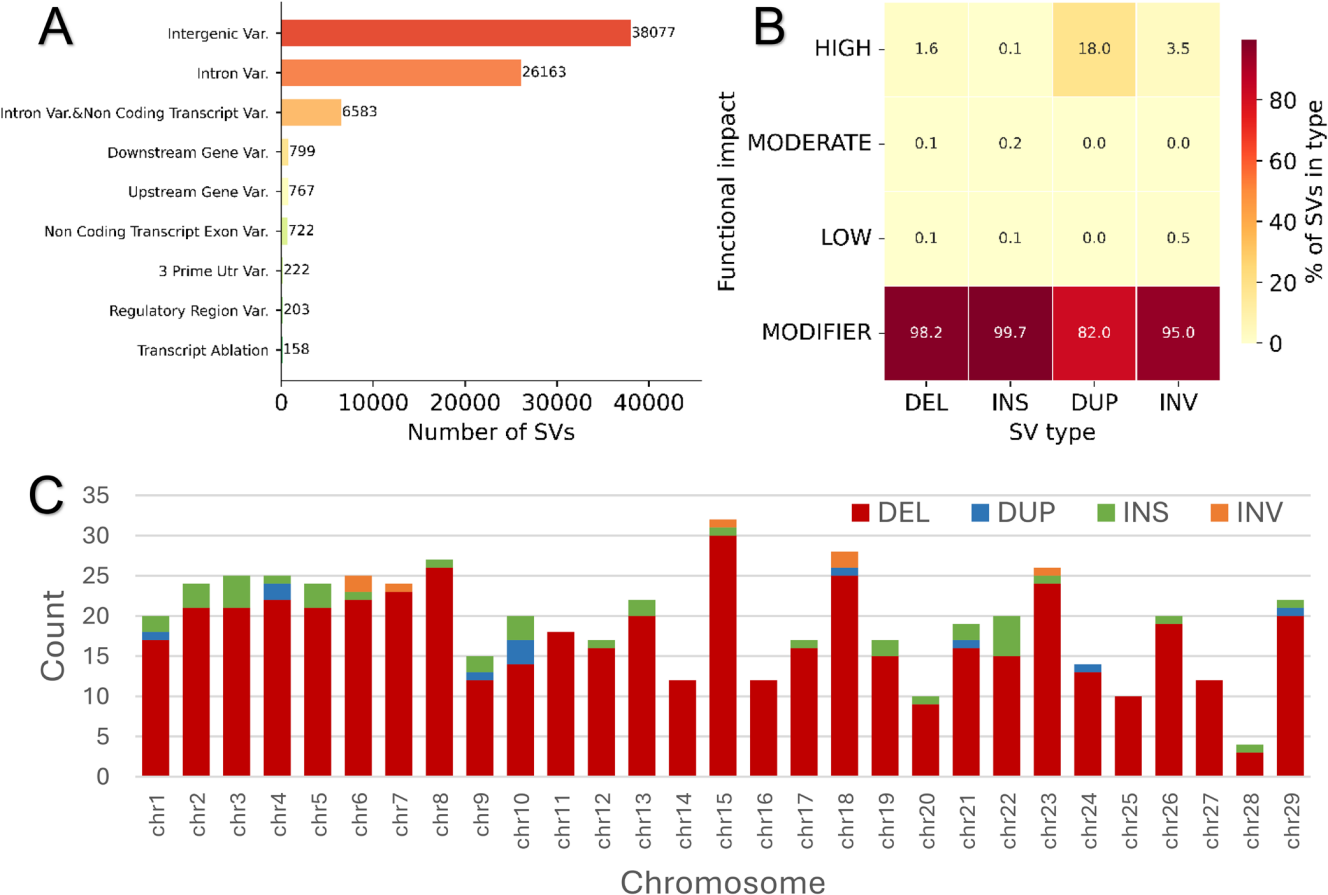
Functional annotation with Ensembl VEP. (A) Number of SVs assigned to each predicted VEP consequence category. (B) Heatmap of the percentage of SVs of each type (columns: DEL, INS, DUP, and INV) falling into each VEP impact tier (rows: HIGH, MODERATE, LOW, and MODIFIER). (C) Number of HIGH‐impact SVs per chromosome, grouped by SV type.

### 3.4 Tag SNP Identification via LD With BovineHD Chip Positions

Pairwise LD analysis between the 74 483 read‐validated SVs and BovineHD SNPs within 100 kb of each SV breakpoint revealed that 22 894 SVs (30.7% of the catalog) were tagged by at least one chip SNP at *r* ^2^ ≥ 0.8 (Figure 4). The remaining 51 589 SVs (69.3%) had no qualifying tag SNP within the defined window. Among the 561 HIGH‐impact SVs, 147 (26.2%) had an assigned tag SNP, whereas 414 (73.7%) did not.

**FIGURE 4:**
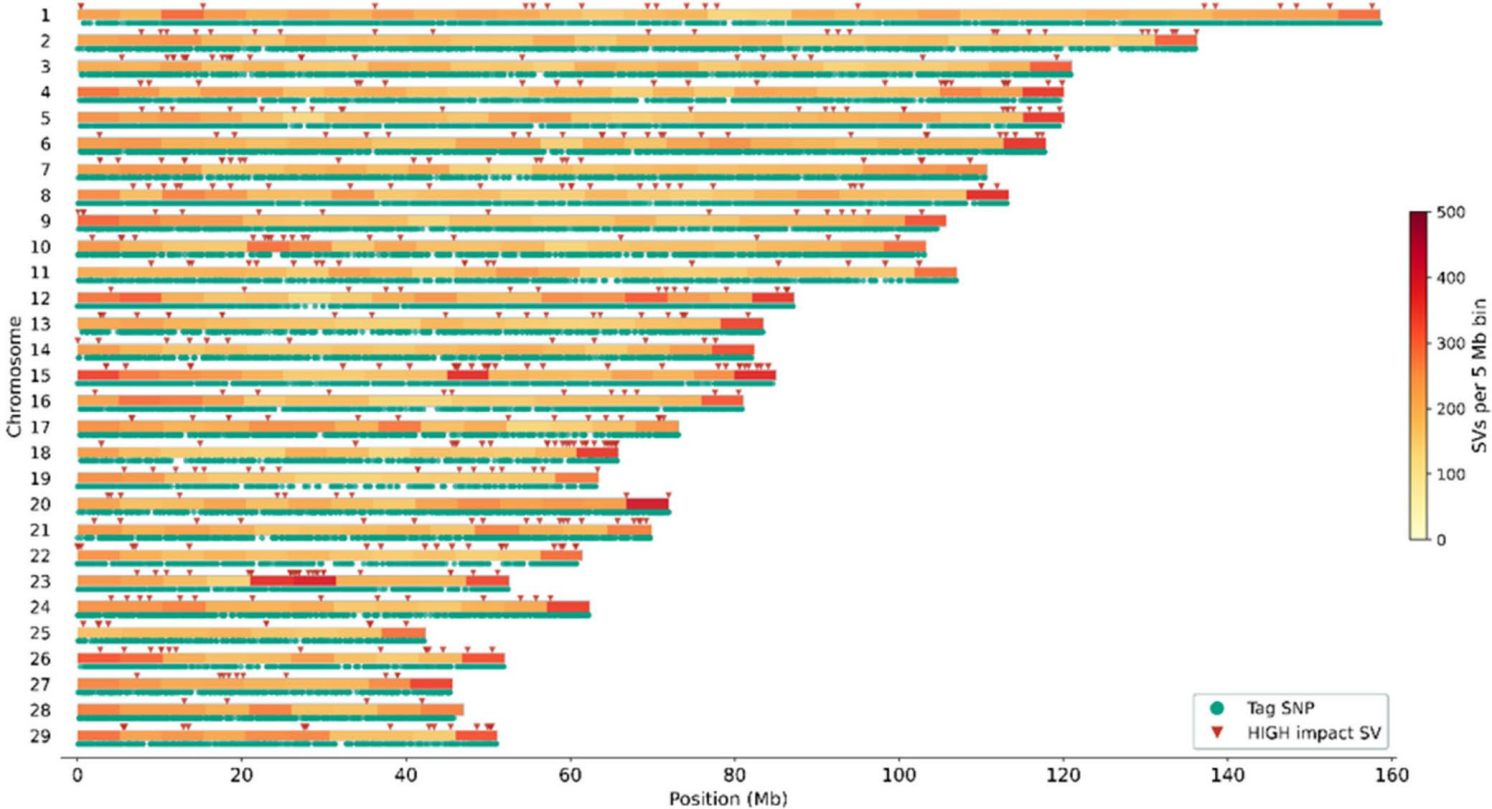
Genome‐wide positions across the 29 autosomes (x‐axis, Mb) of SVs tagged by a BovineHD SNP at *r* ^2^ ≥ 0.8 within 100 kb (teal circles, “Tag SNP”) and of HIGH‐impact SVs (red downward triangles). The background color scale indicates the number of SVs per 5 Mb bin, from pale yellow (low density) to dark red (up to 500 SVs per bin).

The ability of BovineHD chip SNPs to tag SVs through LD varied across SV types (Figure 5A). Deletions showed the highest tagging rate (31.3%), followed by insertions (30.5%) and inversions (2.5%), while no duplication was successfully tagged. The median distance between a tag SNP and its corresponding SV was 16 kb, and the distribution was strongly right‐skewed, with the highest density of pairs concentrated within the first 3 kb (Figure 5B). Only 3 of the 9912 tagged deletions (0.03%) had their tag SNP inside the deleted interval, and none of them was a HIGH‐impact SV. The complete catalog, integrating SV identity, tag SNP identifiers, *r* ^2^ values, annotated gene symbols, and predicted functional impacts, is provided in Data S1.

**FIGURE 5:**
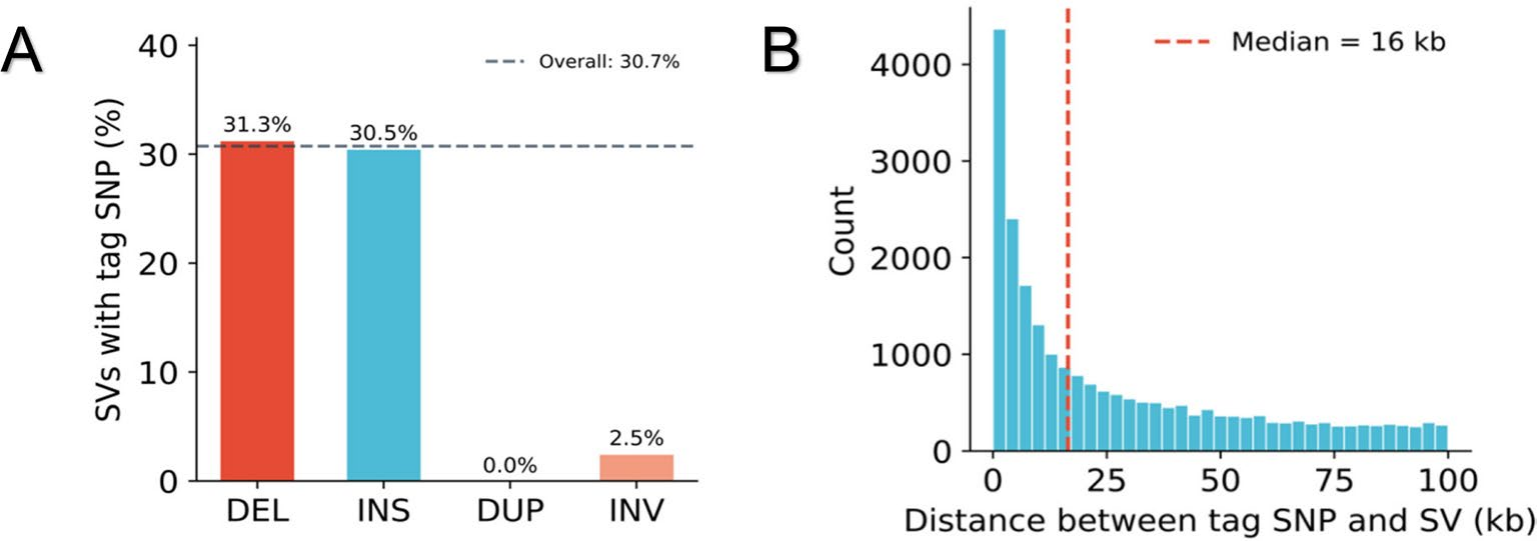
Tagging of read‐validated SVs by BovineHD SNPs. (A) Percentage of each SV type with at least one SNP; (B) Distribution of the physical distance between each tagged SV and its tag SNP.

### 3.5 Indicine‐Specificity of the SV Catalog

Intersection of SV loci with the *Bos indicus* ancestry SNP map derived from Kasarapu et al. (2017) identified 6114 SVs (8.2% of the catalog) as indicine‐enriched (flanking SNPs with Pr_Indicine > 0.5), collectively overlapping 1580 unique indicine‐enriched SNPs (95.6% of the 1652 filtered indicine SNPs) (Figure 6). The highest absolute counts of indicine‐enriched SVs were observed on BTA8, BTA13, and BTA2, with the complete chromosome‐level breakdown in Figure 6. Of the 561 HIGH‐impact SVs in the catalog, 61 (10.87%) overlapped indicine‐specific SNPs, comprising 54 deletions, 6 insertions, and 1 inversion, identifying this subset as the highest‐priority candidates for functional follow‐up. Indicine‐enriched SVs were dominated by insertions and deletions (INS: 3370, 55.1%; DEL: 2723, 44.5%; duplications and inversions were rare, *n* = 4 and 17, respectively) and were markedly small, ranging from 51 bp to 31 054 kb (median: 143 bp), with 80% falling within the 50–500 bp size class.

**FIGURE 6:**
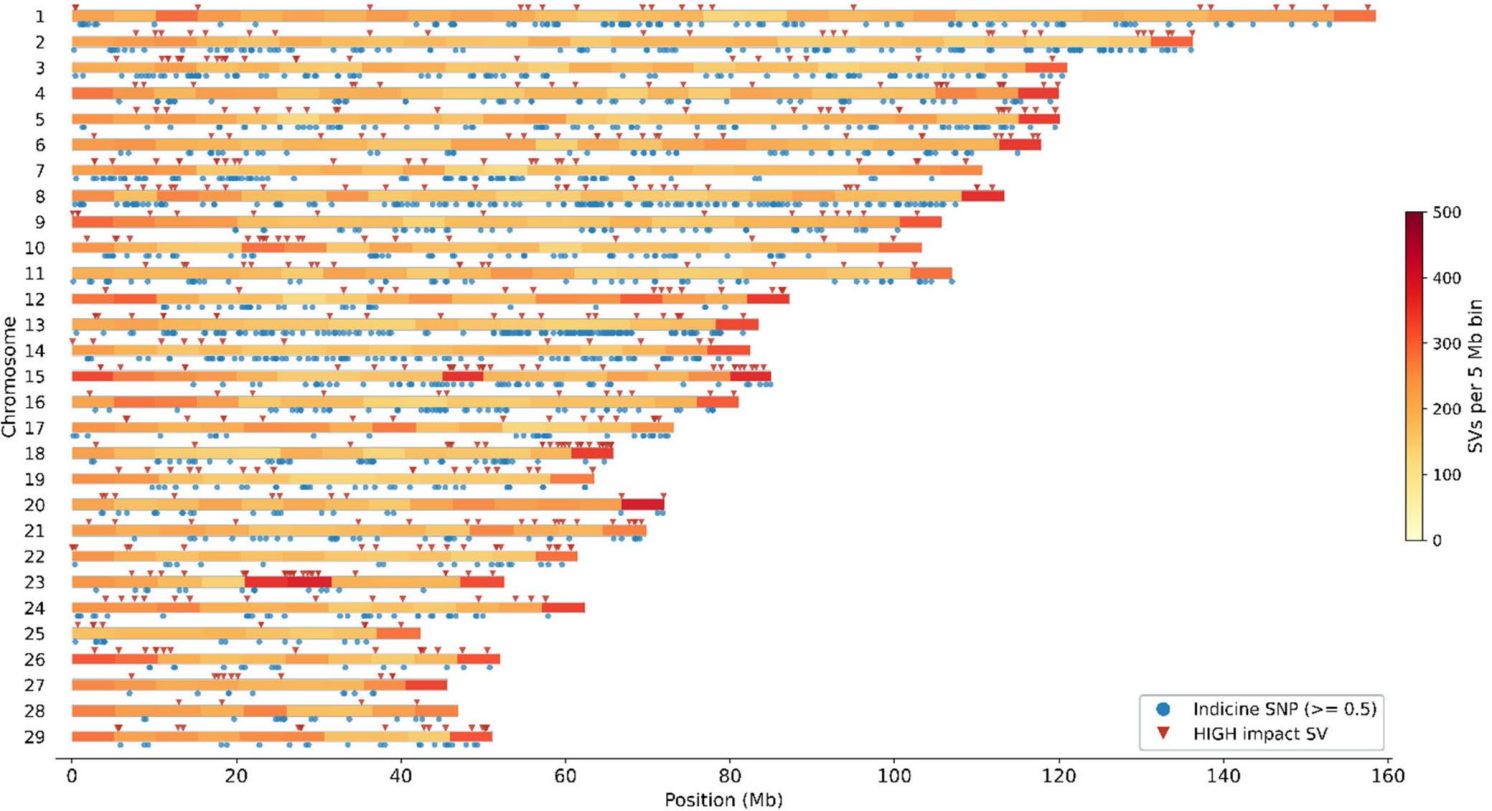
Chromosomal distribution of the 6114 Nelore SVs tagged by SNPs with Pr_Indicine > 0.5 across the 29 autosomes.

Among genes with a defined symbol, *GLB1L3* (BTA15, *n* = 23), *FAM19A5* (BTA5, *n* = 21), and *PCGF3* (BTA5, *n* = 21) were the most frequently overlapped by SVs enriched in *Bos indicus* (complete gene‐level counts in Data S2). Several computationally predicted or uncharacterized loci overlapped even more frequently, most notably *LOC537387* (BTA11, *n* = 30).

## 4 Discussion

A dual‐caller ONT pipeline applied to 20 Nelore bulls yielded a population‐level catalog of 74 483 read‐validated SVs spanning all 29 autosomes, exploiting the superior breakpoint resolution that long‐read approaches provide over short‐read data (Paivandy et al. 2026), to our knowledge the first cohort‐scale SV resource from long‐read sequencing in a purebred indicine breed, extending prior long‐read assembly and pangenome work conducted in taurine and mixed taurine‐indicine genomic contexts (Bortoluzzi et al. 2024; Leonard et al. 2022, 2023) to a population‐level catalog. Despite seminal protein contamination reducing ONT adapter binding efficiency (Denis et al. 2024), the mean sequencing depth of 22.11× was sufficient for population‐scale SV discovery. In addition to the catalog scale, three analytically distinct findings carry specific implications:

- the behavior dependent on the type of SV callers and validation tools, particularly the complete failure of read‐based validation to confirm duplications and inversions;
- the limited, but tractable, coverage of SVs by BovineHD chip markers, with most HIGH‐impact SVs remaining without associated markers; and
- the identification of 6114 enriched SVs in indicine cattle overlapping genes plausibly related to tropical adaptation.

Taken together, these results establish a genomic resource that expands the scope of variant detection in indicine cattle breeds far beyond what has been achievable with short‐read approaches.

### 4.1 A Dual‐Caller, Read‐Validated SV Catalog From ONT Long‐Read Data

The application of two complementary long‐read SV callers, Sniffles2 and SVIM, followed by concordance‐based filtering, produced a population‐level catalog of read‐validated SVs spanning all 29 autosomes. This approach addresses a well‐recognized limitation of individual callers, which are known to produce high false‐positive rates when operated in isolation (Gao et al. 2022; Liu 2025). The dual‐caller intersection retained only variants independently supported by both tools, and subsequent read‐based validation with SVvalidation further reduced false discovery by requiring read‐level evidence in at least two independent animals. This layered quality framework is consistent with recommendations from comparative benchmarking studies showing that multi‐caller concordance substantially increases specificity without sacrificing recall for the most common SV classes (Gao et al. 2022).

We acknowledge that “read‐validated” refers throughout to SVvalidation‐based classification (homozygous or heterozygous versus false positive) using the same ONT read data as SV discovery, not orthogonal experimental confirmation. No PCR or alternative‐platform verification was performed. Cross‐sample corroboration (≥ 2 independent animals) provides a degree of replication that reduces the likelihood of sample‐specific artifacts, but platform‐wide systematic biases shared across ONT libraries would not be detected by this framework. The contrasting read‐validation rates observed across SV types are consistent with the known technical characteristics of split‐read callers. Insertions achieved the highest read‐validation rate (98.71%), which aligns with SVIM's documented advantage in resolving novel element insertions through detection of complementary split‐read signatures not captured by Sniffles2 (Heller and Vingron 2019). Deletions initially showed a substantially lower rate (37.33%) than insertions when assessed by SVvalidation alone. Size‐stratified analysis showed this was concentrated in DELs < 500 bp, where SVvalidation's reliance on an explicit deletion (D) operation in the CIGAR string is prone to under‐detection because long‐read aligners frequently represent short deletions in repetitive or homopolymer‐rich regions as mismatches or soft‐clipped ends rather than explicit D operations (Li 2018). Applying an alternative, read‐support‐based criterion (Sniffles2 DV field, ≥ 5 supporting reads in at least two animals) to this size class recovered the large majority of these variants, raising the overall DEL validation rate to 81.4% and confirming that the original low rate reflected an alignment‐representation artifact rather than an inherent unreliability of DEL calls. DELs ≥ 5 kb validated at 96.3%, fully comparable to insertions.

The absence of read‐validated duplications and inversions after the concordance step is not unexpected: these SV classes consistently show lower inter‐caller agreement and are particularly sensitive to coverage depth and alignment artifacts, as previously documented in cattle (Gao et al. 2022) and humans (Heller and Vingron 2019). The low concordance rates detected at the per‐sample filtering step for DUP (12.6%) and INV (37.4%) suggest that the discordance was intrinsic to the calling signatures, rather than a product of the SVvalidation step itself. This finding reinforces the position that duplications and inversions in ONT data at mean depths of ~22× require assembly‐based or targeted approaches to be reliably resolved (Liu 2025). The absence of these classes in the final catalog represents a gap in the coverage of SV types, categories known to carry high functional potential in mammals (Collins and Talkowski 2025), and limits the scope of this resource to read‐validated insertions and deletions.

This size profile is consistent with patterns reported in long‐read SV studies, where insertions in the 50–500 bp range are predominantly attributed to mobile element activity (Zhou et al. 2022). However, no dedicated transposable element annotation was performed in the present study, and the assignment of the small‐insertion enrichment and of these size modes to specific TE families (SINE, LINE, or LTR) was not tested. At the population level, the modest predominance of insertions over deletions in the indicine‐enriched subset (55.1% INS vs. 44.5% DEL) likely reflects, in part, the underrepresentation of the Nelore genome in the Hereford‐derived ARS‐UCD2.0 reference. Sequences present in the indicine genome but absent from the reference would be captured as insertions relative to that linear scaffold. This interpretation is consistent with prior observations, themselves conditioned on a taurine reference frame, that indicine individuals carry proportionally more SVs than their taurine counterparts (Talenti et al. 2022; Zhou et al. 2022), and that indicine genomes contribute disproportionately to non‐reference unique sequences in multi‐assembly pangenome analyses (Zhou et al. 2022). This asymmetry implies, however, that fixed or nearly fixed SVs in Nelore relative to the Hereford reference will be systematically captured as insertions, whose true biological context as conserved indicine sequences remains unresolved without a Nelore reference mount.

### 4.2 Functional Distribution of SVs: Intergenic Predominance and Autosomal HIGH‐Impact Subset

Ensembl VEP annotation assigned functional consequences to all 74 483 variants relative to 7040 protein‐coding genes. The predominance of intergenic (51.12%) and intronic (35.13%) annotations is consistent with the distribution reported in other cattle SV catalogs (Talenti et al. 2022; Zhou et al. 2022). This pattern is expected because coding sequences represent only a small fraction of the genome, and because SVs that disrupt coding sequences are more likely to be deleterious and therefore kept at low frequency or removed by purifying selection (Zhou et al. 2022). This predominance should not be read as evidence of functional neutrality: intergenic SVs can still disrupt distal regulatory elements such as enhancers and chromatin domain boundaries and have been linked to gene expression variation in cattle and other livestock species, underscoring that VEP's coding‐centric consequence categories do not capture the full functional relevance of this majority class. The 561 SVs classified as HIGH‐impact represent the subset with the greatest predicted disruptive potential, including transcript ablations, frameshifts, and splice site disruptions. This proportion is comparable to the 1.5% high‐impact rate reported in a Holstein catalog generated with the Smoove pipeline (Grant et al. 2024) and to HIGH‐impact SV proportions reported in long‐read cohort analyses of taurine breeds (Mapel et al. 2026), consistent with the expectation that aggressive concordance and validation filtering, as applied here, reduces the proportion of high‐impact calls relative to more permissive pipelines.

The chromosomal concentration of HIGH‐impact SVs is noteworthy. Chromosomes BTA08, BTA15, BTA18, and BTA23 harbor documented QTL regions for immunity‐related and production traits in both taurine and indicine cattle, and recurrent SVs enrichment in these chromosomes may partly reflect their elevated gene density and their immune gene content (Band et al. 1998; Biscarini et al. 2016; Goyache et al. 2022). Grant et al. (2024) noted that the VEP *‐‐pick* flag, which was also used here, conservatively assigns a single consequence per variant and may underestimate the total impact of SVs spanning multiple genes or transcripts, a consideration that applies with force to large SVs overlapping gene clusters. Functional enrichment analysis or multi‐transcript annotation would be required to fully characterize the regulatory and coding impact of the HIGH‐impact subset identified here. An additional annotation asymmetry affects the type composition of the HIGH‐impact subset: VEP annotates SVs against reference coordinates, and deletions span contiguous reference bases that can fully remove exons, disrupt splice sites, or ablate transcripts. Insertions, by contrast, occupy a single‐nucleotide reference footprint regardless of their actual size, limiting VEP's capacity to predict functional consequences for the inserted sequence. Consequently, deletions are expected to be overrepresented among HIGH‐impact predictions despite representing a minority of the overall catalog. The limitation is inherent to reference‐anchored SV effect‐prediction tools and is not specific to this study. SV functional annotation remains an active area of development. More broadly, the HIGH‐impact classification assigned by VEP reflects computational inference of gene consequence and not demonstrated functional effect (McLaren et al. 2016), and phenotypic associations cannot be established from the catalog alone, in the absence of expression data or experimental validation of the predicted disruptions (Lee et al. 2023).

### 4.3 Incomplete and Type‐Dependent Coverage of SVs by BovineHD SNPs

The coverage of the SVs validated by the BovineHD chip is substantially incomplete: only 30.7% of the 74 483 variants were tagged by at least one SNP with *r* ^2^ ≥ 0.8 in a 100 kb window around the breakpoint. This pattern reflects a structural limitation of high‐density array platforms, whose markers were developed mainly from taurine cattle data and were not designed to capture the diversity of SVs (Matukumalli et al. 2009). Ascertainment bias is an additional and likely compounding factor: because the BovineHD chip was designed from taurine populations, only 479 252 of its 777 962 positions (61.6%) were polymorphic in the present Nelore cohort after standard QC filters, confirming that ascertainment bias reduced the effective density of informative markers available to capture LD with indicine‐enriched SVs. In contrast to the tagging rate reported for Holstein cattle (21%; Grant et al. 2024), the higher rate observed here in Nelore (30.7%) indicates that ascertainment bias, while still measurable, did not prevent the BovineHD chip from capturing a substantial fraction of SVs in this indicine population. The wider 100 kb search window applied here relative to prior studies likely also contributed to the higher tagging rate. The scarcity of chip SNPs within short physical distance of SV breakpoints nonetheless remains the fundamental constraint shared across breeds. The tagging rate was dependent on the type of SV: deletions had the highest coverage (31.3%), followed by insertions (30.5%) and inversions (2.5%), while no duplicates reached the *r* ^2^ threshold, reflecting the absence of these variants in the catalog after concordance filtering. This result is directionally consistent with findings from Holstein cattle, where the BovineHD chip captured approximately 21% of SVs detected with a long‐read‐compatible caller (Grant et al. 2024), and with reports from a Holstein Friesian family cohort in which array‐based 50 K SNPs captured only 13.4% of WGS‐detected deletions (Lee et al. 2023). The observed decay of LD with physical distance, with the highest density of SV‐tag SNP pairs concentrated within the first 3 kb, indicates that proximity to the breakpoint is the dominant determinant of tagging success, consistent with the rapid LD decay reported in cattle populations (Lee et al. 2023).

The practical consequence is that 69.3% of the SV catalog, including 73.7% of HIGH‐impact SVs, cannot be captured by routine BovineHD genotyping. While the 22 894 tagged SVs provide an immediately deployable resource for association studies in populations with existing array data, the untagged majority, particularly the 414 HIGH‐impact SVs without tag SNPs, represent a gap that cannot be closed without either direct SV genotyping or imputation from a reference panel. The median physical distance of 16 kb between a tagged SV and its best tag SNP is broadly consistent with the linkage disequilibrium structure in Nelore cattle and suggests that the tagging rates reported here may be replicable across populations with similar ancestry (Espigolan et al. 2013; McKay et al. 2007). Imputation‐based approaches using the present catalog as a reference panel, analogous to strategies applied in French dairy breeds (Naji et al. 2025), represent a practical route to extending SV genotyping via existing BovineHD array data for the 22 894 tagged SVs (30.7% of the catalog). The untagged majority, including 73.7% of HIGH‐impact SVs, cannot be recovered by SNP‐based imputation and would require WGS‐based reference panels or direct SV genotyping for population‐scale deployment. The robustness of this panel, however, will be contingent on expanding the sequencing cohort beyond the 20 bulls analyzed here, since reliable estimates of allele frequency and the inclusion of low‐frequency variants (MAF < 0.05) require substantially larger samples (Liu 2025; Naji et al. 2025).

### 4.4 Genomic Signatures of Tropical Adaptation in Enriched SVs in *Bos indicus*

The overlay of the SV catalog with the *Bos indicus* ancestry probability map by Kasarapu et al. (2017) reveals a genomic signal consistent with differential selection between *Bos taurus* and *Bos indicus* : 6114 SVs enriched in indicines collectively overlap with 1580 unique SNPs (95.6% of the 1652 filtered indicine SNPs) with a posterior probability of origin in *Bos indicus* greater than 0.5. These variants, distributed across all 29 autosomes (highest concentration in BTA8, BTA13, BTA2, BTA1, and BTA3) and dominated by short insertions (50–500 bp), outline a profile consistent with the architecture of indicine SVs described in bovine pangenome analyses (Dai et al. 2023; Talenti et al. 2022). Although inaccuracies in the UMD3.1 coordinate liftover to ARS‐UCD2.0 and the possible ascertainment bias of ancestry SNPs relative to the taurine reference may have affected Pr_Indicine estimates for a subset of loci, the autosome‐wide distribution and the predominance of short insertions among indicine‐enriched SVs suggest that this signal reflects genuine *Bos taurus* – *Bos indicus* differentiation rather than technical artifact. We emphasize that this assignment reflects the ancestry of the haplotype background tagged by flanking ancestry‐informative SNPs rather than direct evidence that each SV allele arose in *Bos indicus* , and that mapping against the taurine ARS‐UCD2.0 assembly necessarily under‐ascertains indicine‐specific sequence. The indicine‐enriched set reported here is therefore best interpreted as a conservative, LD‐anchored subset of the true indicine SV landscape.

Functional interpretation is most informative for the 61 HIGH‐impact SVs that overlap indicine‐enriched loci (54 deletions, 6 insertions, and 1 inversion), where the predicted consequence gives higher confidence that the overlapped gene is functionally affected. Among the 62 gene symbols annotated across these variants, the largest coherent functional block encodes immune and inflammatory mediators, including *PLA2G2A* (secreted phospholipase A2, bactericidal activity at mucosal and epithelial surfaces) (Murakami et al. 2023), *LYZ1* (lysozyme, innate antibacterial defense) (Ragland and Criss 2017), *RIPK2* (NOD‐like receptor signaling downstream of bacterial peptidoglycan recognition) (Nachbur et al. 2015), *BCL11B* (T‐lymphocyte lineage commitment) (Li et al. 2010), and the apoptosis‐associated genes *CNTFR* and *CASP6*. This immune‐inflammatory enrichment parallels the well‐documented superiority of *Bos indicus* over *Bos taurus* in resistance to ticks and tick‐borne pathogens, attributed largely to a more effective innate and cellular immune response (Piper et al. 2009), and mirrors the enrichment of immune‐related genes reported in other SV and CNV studies differentiating the two subspecies (Zhou et al. 2022), including in Gyr cattle, where immunity genes such as *GBP2*, *GBP4*, *BoLA‐DQA1*, and *HERC2* were recurrently found in CNV‐variable regions (Braga et al. 2023).

A second, smaller functional module implicates oxidative‐stress defense (*GSTM1*, *MSRA*; discussed below). Among the individual loci overlapped by HIGH‐impact, indicine‐enriched SVs, *GSTM1* and *MSRA* stand out as a functionally coherent pair. *GSTM1* encodes a phase II detoxification enzyme that conjugates glutathione to reactive electrophiles and oxidative byproducts (Hayes et al. 2005), while *MSRA* repairs oxidatively damaged methionine residues in proteins and scavenges reactive oxygen species (Weissbach et al. 2005). Joint enrichment at these two loci is consistent with a coordinated oxidative‐stress‐response module that may be relevant to the elevated thermal and radiative load of tropical environments, although neither gene's contribution to heat tolerance has been directly tested in cattle. Two deletions within this HIGH‐impact, indicine‐enriched set, affecting *SENP1* on BTA5 and *SHCBP1* on BTA18, represent additional priority candidates for functional follow‐up. *SENP1* encodes a SUMO‐specific protease involved in the sumoylation pathway, which modulates transcriptional responses to hypoxia and oxidative stress, suggesting a potential mechanistic link to heat tolerance (Cheng et al. 2007). *SHCBP1* participates in SHC‐mediated signaling downstream of growth factor receptors, and structural disruption of this locus may influence cell proliferation responses under thermal stress conditions (Peng et al. 2016). These inferences are speculative in the absence of expression or functional data, but the presence of HIGH‐impact SVs at indicine‐enriched loci with plausible roles in immune defense and thermal/oxidative stress response pathways provides a testable hypothesis framework for future validation.

Lineage‐specific SV enrichment in immune and metabolic gene families in indicine breeds has been documented by Bickhart et al. (2016) using CNV analysis across taurine and indicine populations, where genes such as *MCM4* and *KRTAP9‐2* showed clear copy‐number stratification between *Bos taurus* and *Bos indicus* , with the latter gene previously reported as potentially involved in tick resistance in indicine cattle. The indicine‐enriched SV catalog presented here extends these observations to the SV landscape in a purebred Nelore population and, importantly, anchors them to long‐read‐confirmed variant calls rather than array‐inferred copy‐number states, providing higher breakpoint resolution.

The remaining overlapped genes span structural and cytoskeletal (*COL2A1*, *CDH26*, and *DOCK5*), signaling and ubiquitination (*FBXW11*, *STK10*, *PPP2R5C*, *PPP3CC*, and *SORBS3*), reproductive and meiotic (*SUN5*, *SYCP2*, and *ADRA1D*), and genomic maintenance or RNA‐processing (*SMC2*, *FBXO18*, *EWSR1*, *RPRD1A*, *ELP2*, *NSUN2*, *TSEN15*, and *NUMBL*) functions, none of which have an established connection to thermal or tropical adaptation. Two caveats temper these functional assignments: approximately 23% of the overlapped loci are uncharacterized LOC genes with no annotated function, and every SNP‐gene overlap in this set is supported by only one or two ancestry‐informative markers, so these annotations should be treated as hypothesis‐generating rather than as validated biological signal.

## 5 Conclusions

The Nelore SV catalog reported here establishes a breed‐specific, long‐read‐validated resource that advances the genomic characterization of the most numerically important indicine breed in the world. The identification of 22 894 SVs (30.7% of the catalog) with BovineHD tag SNPs provides an immediately applicable panel for SV‐informed GWAS and genomic selection in populations with existing array data, though the remaining 69.3% of the catalog, including 73.7% of HIGH‐impact SVs, falls outside current chip coverage and will require whole‐genome sequencing or imputation‐based approaches for population‐scale use, while the 6114 indicine‐enriched SVs, including 61 HIGH‐impact variants at loci with plausible roles in immune/inflammatory defense and oxidative‐stress response, define candidate variants for follow‐up association studies targeting disease resistance and thermotolerance traits. Integrating this catalog into breeding pipelines to complement SNP‐based genomic evaluations and validating the HIGH‐impact indicine‐enriched SVs through expression quantitative trait locus analyses and phenotype association studies in large Nelore cohorts, represents the most direct path toward translating these findings into genomic selection for tropical adaptability.

## Funding

This work was supported by Fundação de Amparo à Pesquisa do Estado de São Paulo, 2021/03101‐9, 2023/06637‐2, and 2024/18544‐1.

## Conflicts of Interest

The authors declare no conflicts of interest.

## Supporting information


**Table S1:** List of bulls selected for the study based on information and genetic diversity.
**Table S2:** Genome size (autosomes only), data amounts, and read depth per sample.
**Table S3:** Chromosome‐wise counts of heterozygous and homozygous deletion and insertion calls across the Nelore cohort.
**Table S4:** Structural variants validation success rate by SV type and size class (support ≥ 2 animals out of 20).

**Data S1:** Structural variants (SVs) identified across the reference genome using SVIM, annotated with predicted functional consequences, and cross‐referenced with linked tag SNPs. N_AMOSTRAS_VALIDADAS and AMOSTRAS indicate the number and identity of samples in which each SV was validated; HAS_TAG_SNP flags SVs with an associated tag SNP.

**Data S2:** Structural variants (SVs) detected across cattle genomes, with genomic coordinates, SV type and length, caller/sample support, overlap with zebu ( *Bos indicus* )‐ and taurine ( *Bos taurus* )‐informative SNPs, ancestry probability estimates, and annotation of overlapping genes.

## Data Availability

The data that support the findings of this study are openly available in Data for Nelore Cattle Structural Variant Catalog at https://zenodo.org/records/21878484, reference number DOI: 10.5281/zenodo.21878484.
