# Supplementary material for: A Nelore Cattle Structural Variant Catalog
Source: Anim Genet. 2026 Oct 18;57(5):e70225. doi: 10.1002/age.70225 (PMC13677160; doi:10.1002/age.70225)
Supplement: Supplementary file 1 [file AGE-57-0-s002.docx]

**A Nelore Cattle Structural Variant Catalog**

**______________________________________________________________________**

Supplementary Information

**______________________________________________________________________**

FAPESP - Grants #2021/03101-9, #2023/06637-2 and #2024/18544-1

Thales de Lima Silva et al.

Pirassununga – SP

2026

General Information

- Title:

**A Nelore Cattle Structural Variant Catalog**

- Research team:

**Thales de Lima Silva¹^,^*, Alexander S. Leonard², Pablo Augusto de Souza Fonseca³, Haroldo Henrique de R. Neves^4^, Gerardo A. Fernandes Júnior^5^, Alexandre M. Ruiz¹, Lucas Tassoni Andrietta¹, Júlio Cesar de C. Balieiro¹, Hubert Pausch², Roberto Carvalheiro^6,7^, and Ricardo Vieira Ventura¹**

- Main institution:

**¹Department of Animal Nutrition & Production, School of Veterinary Medicine and Animal Science, University of São Paulo, Pirassununga, Brazil.**

- Collaborating institutions

**^2^Animal Genomics, ETH Zürich, Zürich, Switzerland. ^3^Instituto de Ganadería de Montaña, CSIC – Universidad de León, Grulleros, León, Spain. ^4^GenSys Consultores Associados, Jaboticabal, Brasil. ^5^Department of Animal Science, Universidade Estadual Vale do Acaraú, Sobral, Brazil. ^6^Department of Animal Science, School of Agricultural and Veterinarian Sciences, São Paulo State University, Jaboticabal, Brazil. ^7^Commonwealth Scientific and Industrial Research Organization, Hobart/TAS, Australia.**

- Corresponding author:

***tl.silva@usp.br**

- FAPESP grants:

**#2021/03101-9, #2023/06637-2 and #2024/18544-1**

SUMMARY

[1.	TABLES	2](#_Toc231884876)

# 1. **TABLES**

## Table 1. List of bulls selected for the study based on information and genetic diversity.

| Name | nCD | nrebD | Rank margc | k10 | Rank margc +k10 | Rank GRM | Final Rank |
| --- | --- | --- | --- | --- | --- | --- | --- |
| COWADAPT_001 | 24,553 | 155 | 1 | 3 | 1 | 1 | 1 |
| COWADAPT_002 | 20,770 | 228 | 7 | 2 | 2 | 4 | 2 |
| COWADAPT_003 | 6,963 | 117 | 26 | 4 | 4 | 7 | 3 |
| COWADAPT_004 | 2,000 | 69 | 8 | 5 | 3 | 10 | 4 |
| COWADAPT_005 | 8,976 | 154 | 18 | 2 | 15 | 3 | 5 |
| COWADAPT_006 | 2,471 | 74 | 52 | 5 | 13 | 5 | 6 |
| COWADAPT_008 | 7,140 | 99 | - | 10 | 10 | 11 | 8 |
| COWADAPT_009 | 15,174 | 108 | - | 6 | 9 | 15 | 9 |
| COWADAPT_010 | 2,012 | 33 | 44 | 8 | 5 | 20 | 10 |
| COWADAPT_011 | 15,203 | 124 | - | 9 | 8 | 40 | 11 |
| COWADAPT_012 | 251 | 18 | - | 1 | 17 | 13 | 12 |
| COWADAPT_013 | 9,685 | 31 | 22 | 2 | 16 | 18 | 13 |
| COWADAPT_014 | 5,751 | 103 | 30 | 2 | 18 | 16 | 14 |
| COWADAPT_015 | 6,186 | 90 | 29 | 4 | 6 | 29 | 15 |
| COWADAPT_016 | 3,198 | 54 | - | 2 | 35 | 2 | 16 |
| COWADAPT_017 | 3,590 | 56 | - | 7 | 12 | 25 | 17 |
| COWADAPT_018 | 7,474 | 80 | 33 | 2 | 19 | 21 | 19 |
| COWADAPT_019 | 5,059 | 90 | 75 | 3 | 23 | 33 | 20 |
| COWADAPT_020 | 10,563 | 123 | - | 2 | 33 | 32 | 22 |
| COWADAPT_023 | 7,771 | 100 | - | 9 | 20 | 6 | 24 |
| nCD = number of offspring at weaning in the Aliança Nelore program; nrebD = number of herds with bull offspring in the Aliança Nelore program; margc = marginal contributions; k10 = partition with 10 groups by hierarchical grouping based on GRM among 2,544 most informative bulls genotyped in the Aliança database. | | | | | | | |

## Table 2. Genome size (autosomes only), data amounts, and read depth per sample.

| **Sample** | **Genome size (bp)** | **Genome data (Gb)** | **Read Depth** |
| --- | --- | --- | --- |
| COWADAPT_001 | 2,770,283,909 | 44,241,297,645 | 15.97 |
| COWADAPT_002 | 2,770,273,741 | 36,865,167,101 | 13.31 |
| COWADAPT_003 | 2,770,563,590 | 60,883,722,333 | 21.98 |
| COWADAPT_004 | 2,769,669,353 | 29,033,925,265 | 10.48 |
| COWADAPT_005 | 2,769,870,259 | 78,520,858,040 | 28.35 |
| COWADAPT_006 | 2,770,345,814 | 43,197,698,679 | 15.59 |
| COWADAPT_008 | 2,769,533,528 | 43,075,404,096 | 15.55 |
| COWADAPT_009 | 2,770,335,027 | 67,849,764,315 | 24.49 |
| COWADAPT_010 | 2,770,105,416 | 80,389,022,028 | 29.02 |
| COWADAPT_011 | 2,770,197,125 | 77,039,904,956 | 27.81 |
| COWADAPT_012 | 2,769,760,566 | 47,005,180,782 | 16.97 |
| COWADAPT_013 | 2,770,401,791 | 60,301,690,133 | 21.77 |
| COWADAPT_014 | 2,770,165,172 | 53,714,814,481 | 19.39 |
| COWADAPT_015 | 2,770,485,524 | 53,286,810,085 | 19.23 |
| COWADAPT_016 | 2,770,203,459 | 52,224,754,539 | 18.85 |
| COWADAPT_017 | 2,770,301,322 | 89,764,617,534 | 32.40 |
| COWADAPT_018 | 2,770,365,123 | 43,980,590,738 | 15.88 |
| COWADAPT_019 | 2,770,241,765 | 109,454,552,226 | 39.51 |
| COWADAPT_020 | 2,770,034,801 | 90,114,195,829 | 32.53 |
| COWADAPT_023 | 2,770,436,467 | 63,791,693,620 | 23.03 |

## Table 3. Chromosome-wise counts of heterozygous and homozygous deletion and insertion calls across the Nelore cohort.

| Chr | heter DEL | homo DEL | heter INS | homo INS | Total |
| --- | --- | --- | --- | --- | --- |
| 1 | 13,067 | 6,423 | 25,640 | 4,856 | 49,986 |
| 2 | 10,392 | 4,705 | 18,801 | 3,876 | 37,774 |
| 3 | 9,305 | 3,774 | 16,028 | 3,028 | 32,135 |
| 4 | 10,895 | 4,881 | 19,523 | 3,877 | 39,176 |
| 5 | 9,717 | 5,309 | 18,350 | 3,514 | 36,890 |
| 6 | 10,844 | 4,775 | 18,397 | 3,403 | 37,419 |
| 7 | 9,002 | 3,633 | 16,970 | 2,950 | 32,555 |
| 8 | 9,090 | 4,141 | 17,122 | 3,332 | 33,685 |
| 9 | 8,980 | 4,382 | 16,201 | 3,059 | 32,622 |
| 10 | 8,453 | 4,044 | 15,387 | 2,692 | 30,576 |
| 11 | 6,902 | 3,998 | 15,368 | 3,166 | 29,434 |
| 12 | 8,691 | 4,282 | 16,588 | 2,937 | 32,498 |
| 13 | 5,861 | 3,005 | 10,664 | 2,251 | 21,781 |
| 14 | 6,513 | 2,627 | 11,830 | 2,200 | 23,170 |
| 15 | 9,588 | 3,946 | 17,395 | 2,483 | 33,412 |
| 16 | 6,118 | 3,301 | 12,749 | 2,340 | 24,508 |
| 17 | 6,720 | 2,743 | 11,083 | 2,284 | 22,830 |
| 18 | 5,268 | 2,079 | 8,945 | 1,636 | 17,928 |
| 19 | 4,313 | 1,834 | 8,244 | 1,845 | 16,236 |
| 20 | 6,939 | 4,031 | 13,583 | 2,823 | 27,376 |
| 21 | 5,769 | 2,647 | 11,122 | 2,149 | 21,687 |
| 22 | 4,754 | 1,670 | 8,102 | 1,647 | 16,173 |
| 23 | 6,173 | 2,077 | 10,861 | 1,654 | 20,765 |
| 24 | 5,849 | 3,058 | 10,956 | 2,415 | 22,278 |
| 25 | 3,288 | 1,335 | 5,423 | 1,363 | 11,409 |
| 26 | 5,322 | 2,425 | 9,349 | 1,739 | 18,835 |
| 27 | 5,067 | 2,115 | 9,258 | 1,623 | 18,063 |
| 28 | 4,849 | 2,376 | 9,165 | 1,541 | 17,931 |
| 29 | 5,697 | 2,489 | 10,337 | 1,831 | 20,354 |
| All | 213,426 | 98,105 | 393,441 | 74,514 | 779,486 |

## Table 4. Structural variants validation success rate by SV type and size class (support ≥ 2 animals out of 20).

| Size class (kb) | DEL input | DEL validated | DEL rate | INS input | INS validated | INS rate |
| --- | --- | --- | --- | --- | --- | --- |
| 0.05–0.1 | 10,702 | 603 | 5.6% | 11,803 | 11,629 | 98.5% |
| 0.1–0.2 | 9,320 | 725 | 7.8% | 11,267 | 11,023 | 97.8% |
| 0.2–0.5 | 7,510 | 1,083 | 14.4% | 8,924 | 8,588 | 96.2% |
| 0.5–1 | 4,446 | 1,458 | 32.8% | 4,688 | 4,579 | 97.7% |
| 1–5 | 5,772 | 4,054 | 70.2% | 6,247 | 6,209 | 99.4% |
| 5–50 | 1,175 | 1,132 | 96.3% | 463 | 459 | 99.1% |
